# COVID-19 Infection Presenting With Severe Hydrocephalus and Acute Stroke: A Case Report

**DOI:** 10.7759/cureus.30592

**Published:** 2022-10-22

**Authors:** Omotola Oredipe, Anmol S Johal, Chinwendu Onuegbu, Ayoola Kalejaiye, Chiugo Okoye, Helen Pozdniakova, Ndausung Udongwo, Swapnil V Patel

**Affiliations:** 1 Internal Medicine, University of Lagos College of Medicine, Lagos, NGA; 2 Internal Medicine, Jersey Shore University Medical Center, Neptune City, NJ, USA; 3 Internal Medicine, Montefiore Medical Center, Moses Campus, Bronx, USA; 4 Internal Medicine, Oba Okunade Sijuwade College of Health Sciences, Igbinedion University, Okada, Benin City, NGA; 5 Internal Medicine, Jersey Shore University Medical Center, Neptune City, USA; 6 Internal Medicine, Jersey Shore University Medical Center, Neptune, USA

**Keywords:** headache, covid-19, cerebrospinal fluid, stroke, hydrocephalus

## Abstract

Hydrocephalus is the accumulation of cerebrospinal fluid (CSF) in the cerebral ventricles and is considered an emergency in acute presentation. Hydrocephalus typically presents with symptoms of headache, nausea, vomiting, lethargy, vision changes and seizure; furthermore, narrowing down the underlying etiology of hydrocephalus can aid in treatment and management options. We present a rare case of a patient that presented with a recent diagnosis of COVID-19 and was found to have acute hydrocephalus and stroke. The aim of this case report is to explore the link between COVID-19 and the development of hydrocephalus and stroke by delineating the underlying pathophysiology of COVID-19 as well as the etiologies of hydrocephalus and possible management strategies. We hope to highlight the importance of keeping an open differential for presentations of headaches and also emphasize the potential complications of COVID-19 infection to help better patient outcomes.

## Introduction

Hydrocephalus can present in many different forms and results from the discrepancy in production and absorption of CSF or obstruction that leads to increased intracranial pressure via cerebrospinal fluid (CSF) accumulation in the ventricles of the brain [1]. The diagnosis is made through clinical signs and symptoms on presentation of headache, nausea, vomiting, seizures, lethargy, confusion and other various neurological manifestations [2]. Hydrocephalus can occur at any age and can be the result of a number of different underlying processes such as infection, stroke, hemorrhage, and obstructive tumors among others [3].

Coronavirus disease (COVID-19), caused by the novel coronavirus, severe acute respiratory syndrome coronavirus-2 (SARS-CoV-2, 2019-nCoV), typically affects the respiratory system but can present with manifestations in other systems [4]. Neurological manifestations of COVID-19 such as headache, anosmia and ageusia are commonly present. Other common findings such as stroke, seizures and encephalopathy have also been reported [5]. There have been very few cases reported of hydrocephalus resulting from sequelae of COVID-19 after review of the literature, making this a very rare presentation [6]. We present an interesting case of COVID-19 in a 25-year-old woman presenting with severe hydrocephalus and acute stroke, with a look into the underlying mechanisms of the disease in hopes of forming a connection between recent infection and acute hydrocephalus.

## Case presentation

A 25-year-old woman with no past medical history was brought to the emergency department by the emergency medical services (EMS) due to prolonged vomiting leading to unresponsiveness. The patient tested positive for COVID-19 seven days prior to the presentation and was self-quarantined at home prior to this episode. The night prior to the presentation, the patient experienced sudden onset, moderate to severe right-sided headaches associated with nausea. The headache was minimally relieved by acetaminophen.

Later on, she started to experience severe, prolonged vomiting leading to unresponsiveness which led to the EMS being called. The patient was unresponsive upon the arrival of the EMS and the patient did not experience a fall. On arrival at the emergency department at the outside facility, the patient had a blood pressure of 92/63 mmHg, pulse of 58 beats per minute, respiratory rate of 20 breaths per minute and oxygen saturation of 91% on room air. She remained obtunded and was only minimally responsive to painful stimuli. Laboratory findings including complete blood count and comprehensive metabolic panel were unremarkable. The electrocardiogram showed sinus bradycardia and no evidence of atrial fibrillation. Thyroid stimulating hormone, lipid panel and hemoglobin A1c were all within normal limits.

**Figure 1:**
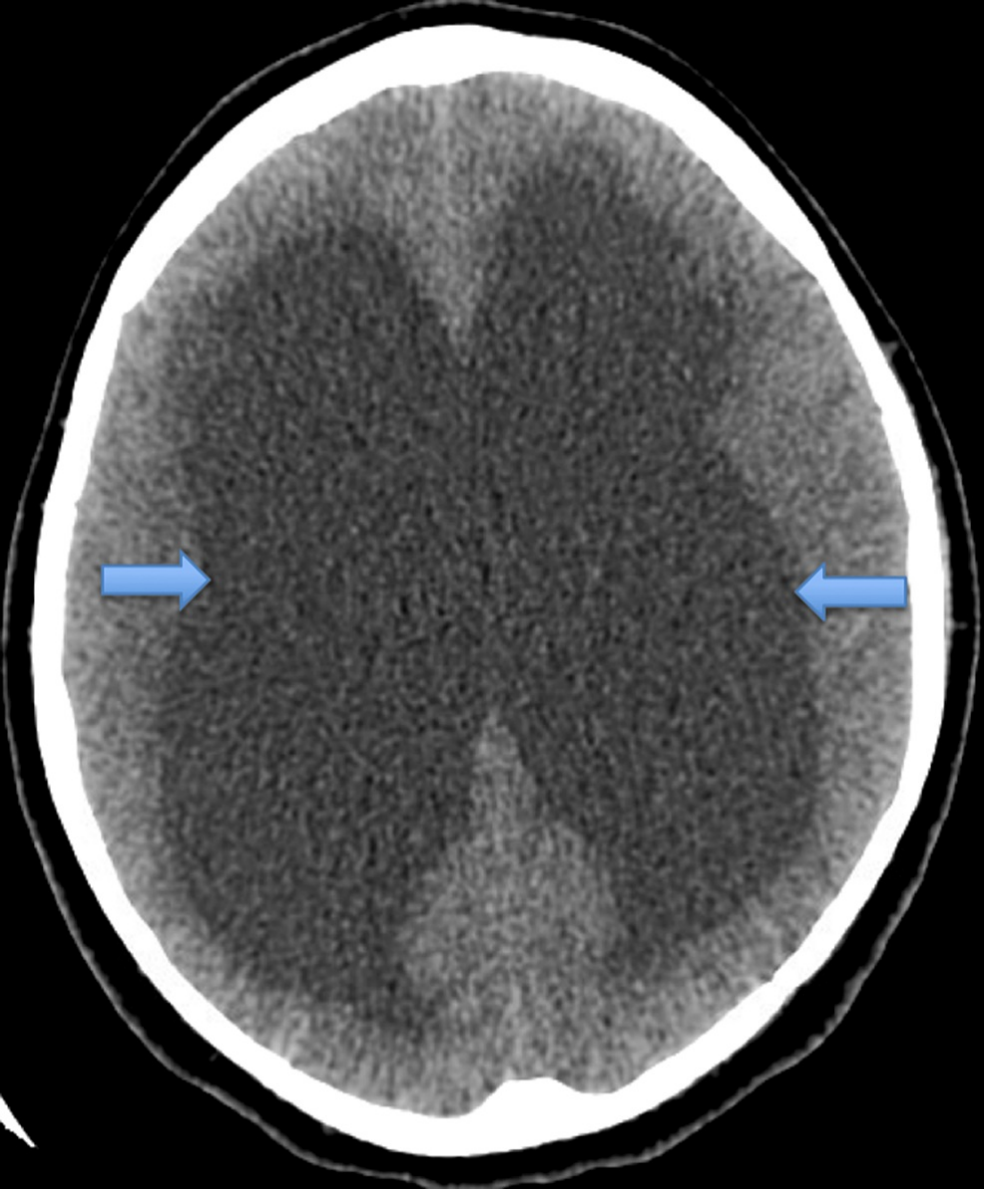
Computed tomography scan of the head without contrast showing acute hydrocephalus involving the lateral (indicated by arrows) and third ventricles, with trans-ependymal flow of CSF. CSF - cerebrospinal fluid

**Figure 2:**
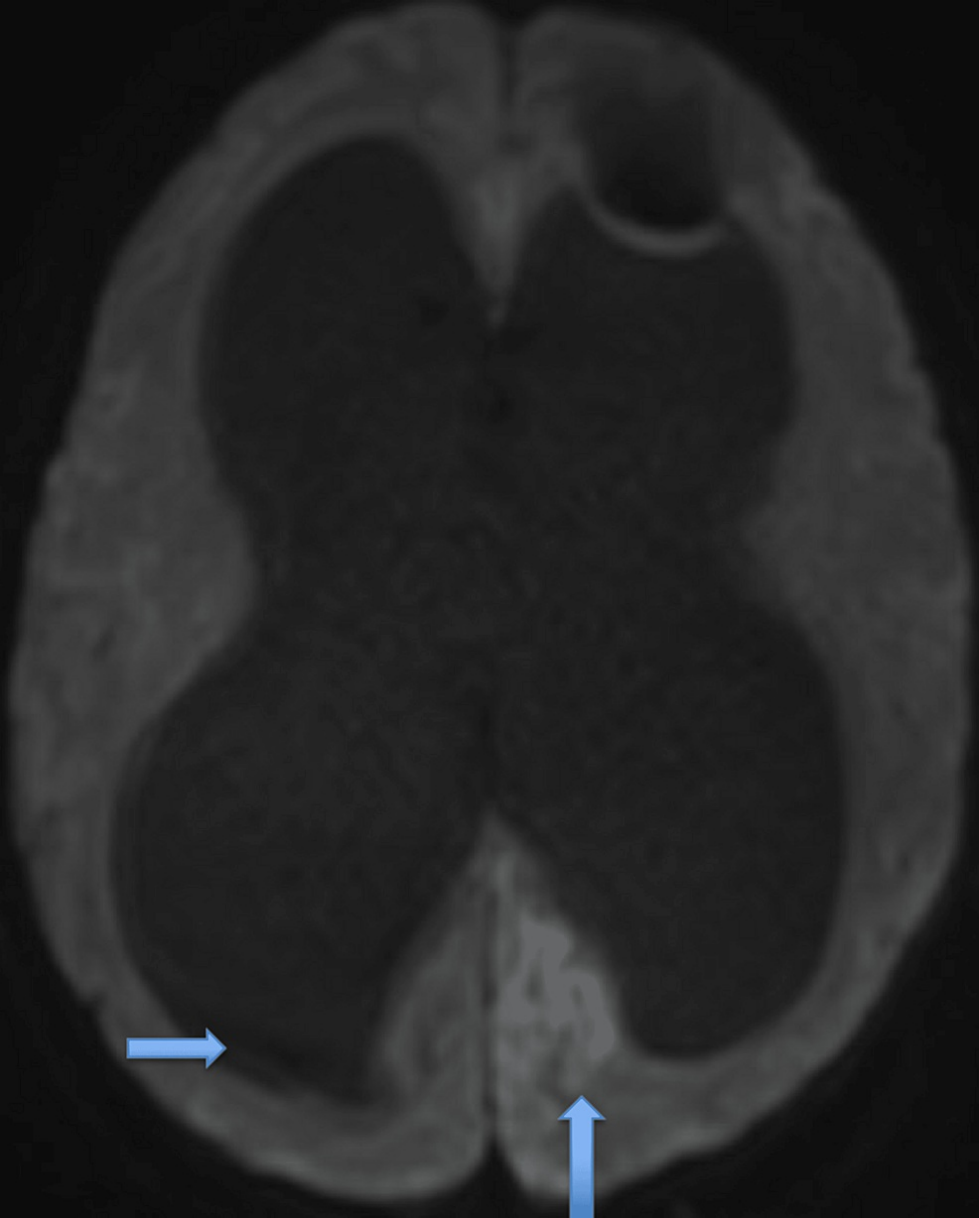
Magnetic resonance imaging of the brain showing multiple acute infarcts in the bilateral thalami and left posterior cerebral artery distribution (upward arrow). Also shows marked ventriculomegaly with trans-ependymal edema (side arrow).

A computed tomography (CT) of the head was done which showed severe obstructive hydrocephalus with dilatation of the lateral and third ventricles and trans-ependymal flow of the CSF (Figure 1), suggesting acute hydrocephalus. No evidence of acute infarct or intracranial hemorrhage was seen. Magnetic resonance imaging (MRI) of the brain was also obtained showing multiple acute infarcts in the bilateral thalami and left posterior cerebral artery distribution, as well as marked ventriculomegaly with trans-ependymal edema (Figure 2). She was given mannitol to reduce intracranial pressure and transferred to our medical intensive care unit (MICU) for urgent neurosurgery evaluation and a higher level of care. Upon arrival at the MICU, the patient was intubated for airway protection and mechanically ventilated. An urgent bedside external ventricular drain was placed. An echocardiogram with bubble study was performed, revealing no atrial or ventricular clots, patent foramen ovale or valvular disease. During the patient's stay in the MICU, she was evaluated for possible etiologies of stroke and transient ischemic attack, metabolic abnormalities, malignancy and syncope were ruled out as potential causes and presentations of stroke. The neurology and stroke teams evaluated the patient and started her on aspirin and high-intensity statin, deep venous thrombosis (DVT) and pulmonary embolism prophylaxis were initiated. Doppler ultrasound of the lower extremities showed no evidence of DVT. Electroencephalography was done and showed no electrographic or clinical seizure-like activity. 

Due to numerous other causes of acute stroke and hydrocephalus being ruled out, it was believed that this was most likely secondary to infection by COVID-19. The patient was able to be transferred from the MICU and was sent to a long-term care facility for prolonged neurological rehabilitation and continued care.

## Discussion

Similar to two other human coronaviruses (CoV), Middle East respiratory syndrome (MERS-CoV) and severe acute respiratory syndrome (SARS-CoV-1) which are known to cause strokes, the SARS-CoV-2 virus has also been widely reported to present with stroke [5]. There are, however, very few reports of hydrocephalus following COVID-19 infection in the literature. Hydrocephalus is the accumulation of CSF in the cerebral ventricles, which may be due to an obstruction to the flow of CSF, excessive CSF production or poor absorption of CSF into the venous system. Symptoms of hydrocephalus include headache, nausea, vomiting, lethargy, irritability and confusion [2]. In this case, the patient presented with severe headache, nausea and vomiting, a week after she had been diagnosed with COVID-19, which after evaluation led to a diagnosis of acute hydrocephalus.

The exact mechanism of hydrocephalus after COVID-19 infection is unclear but proposed explanations are related to the ability of the SARS-CoV-2 virus to bind to human cells via the angiotensin-converting enzyme 2 (ACE2) receptor. The ACE2 receptor is widely expressed in tissues such as respiratory epithelia, kidneys, liver, blood vessels and the brain. The binding of the SARS-CoV-2 virus to the ACE2 receptor triggers the release of cytokines which leads to increased vascular permeability, edema and widespread inflammation in various organs. This inflammatory response also triggers hypercoagulation and the formation of blood clots which can lead to neurological manifestations such as stroke and can lead to impaired venous flow and congestion [7]. Other proposed mechanisms of the neurological findings in COVID-19 infection are viral neuro-invasion via trans-synaptic transfer across infected neurons, direct viral entry via the olfactory nerve and migration of infected leukocytes across the blood-brain barrier [5,8]. There are large concentrations of ACE2 receptors in the choroid plexus of the ventricles and as such, interactions between the SARS-CoV-2 virus and the choroid plexus may possibly lead to alterations in the flow of CSF and result in the formation of arachnoid webs which prevent longitudinal CSF flow [9]. In a study by Alexopoulos et al., it was found that eight of eight patients had anti-SARS-CoV-2 antibodies in their CSF, which could suggest a possible etiology with a combination of blood-brain barrier disruption after infection and cytokine release [10].

Although our patient presented within a week of developing COVID-19 symptoms, there have been reports of hydrocephalus developing up to three months after recovery from COVID-19 infection which suggests that hydrocephalus can develop at any point post-infection with COVID-19 [9]. Headache which is a common symptom of COVID-19 requires careful evaluation due to the possibility of masking severe underlying findings such as hydrocephalus [11]. Acute hydrocephalus is a medical emergency, which as in our patient’s case, is commonly treated by urgent placement of an external ventricular drain. After placement of the external ventricular drain and mannitol, our patient’s symptoms improved. Failure to adequately treat hydrocephalus may lead to complications such as visual changes, temporal lobe herniation, cognitive dysfunction, and gait problems [2].

## Conclusions

Our case aims to highlight the rare presentation of acute hydrocephalus and stroke as a potential complication of recent COVID-19 infection. We explore the various types and mechanisms of hydrocephalus as well as the pathophysiology of COVID-19 and extrapolate a possible pathway of how it can develop into these disease states. Given the case presentation and the rule out of most other major causes of stroke and hydrocephalus, the hypercoagulability secondary to COVID-19 infection could be a possible etiology in this case. There is still little known about the complete mechanism of COVID-19 and what it can ultimately cause in terms of complications and various sequelae of the disease. We hope to add to the understanding of acute hydrocephalus and COVID-19 through this case report and underscore the importance of recognizing recent infections, keeping an open differential and prompt management of medical emergencies.
